# Interventions for environmentally sustainable and climate-resilient cities and communities for the aging population in South Korea: a scoping review

**DOI:** 10.3389/fpubh.2026.1731172

**Published:** 2026-04-13

**Authors:** Hyunjin Kang, Jieun Oh, Ho Kim, Siwon Lee, Mikiko Kanda, Pankyu Park, Sally Edwards

**Affiliations:** 1 Institute of Health and Environment, Seoul National University, Seoul, Republic of Korea; 2 Graduate School of Public Health, Seoul National University, Seoul, Republic of Korea; 3 World Health Organization Regional Office for the Western Pacific, Manila, Philippines; 4 WHO Asia-Pacific Centre for Environment and Health in the Western Pacific Region, Seoul, Republic of Korea

**Keywords:** AFCC, age-friendly cities and communities, climate resilience, environmental sustainability, Korea, older adults, healthy aging, healthy city

## Abstract

**Introduction:**

As the Republic of Korea undergoes demographic transitions, exposure to climate-related health risks is intensifying. In response, the government has considered population aging as part of their national plans of climate adaptation and health, and local governments have also worked on age-friendly city plans aligned with WHO guidelines. However, evidence of interventions contributing to environmental sustainability and climate resilience remains limited and unclear.

**Methods:**

Synthesizing Korean-language evidence, this review is the first to map how age-friendly interventions incorporate or miss climate resilience and environmental sustainability through a scoping review.

**Results:**

Using three Korean search engines (DBpia, KCI, RISS) on June 3, 2024, 35 interventions were retrieved from the final 31 publication records. The review found that interventions for age-friendly cities and communities (AFCC) largely neglect environmental sustainability and climate resilience, with government-driven initiatives relying on limited indicators. The most popular AFCC domains that the reviewed interventions belong to were Domain 1 (Outdoor spaces and buildings), followed by 8 (Community support and health services) and 7 (Communication and information). No interventions were observed under Domain 5 (Civic participation and employment).

**Discussion:**

Capturing limited comprehensiveness, predominance of government-driven interventions, and lack of robust evidence on their effectiveness, this review underscores the need to integrate climate change considerations into the under-addressed domains of the AFCC framework. At the policy level, the authors recommend promoting young older adults’ community engagement, innovation through non-governmental sector, and development of indicators through implementation research.

## Introduction

The Republic of Korea (hereinafter Korea) is undergoing an unprecedented pace of demographic transition. The number of older individuals aged 65 or older in Korea is estimated to continue to increase from 18.4% of the total population (9.5 million) in 2023 to 30.1% (15.3 million) in 2035 (1). In particular, Korea is expected to enter an aged society in 18 years and a super-aged society 7 years thereafter. This demographic shift is occurring at a more rapid pace compared to Japan (25 and 10 years) and the United States (72 and 15 years) (1). At the same time, Korea is undergoing rapid climate change. The average annual temperature, sea-level temperature and sea level rise in Korea are faster than the global average. The average annual temperature in Korea has increased by about 1.6 °C over the past 109 years (1912–2020), the sea-level temperature by 1.23 °C over the past 50 years (1968–2017), and sea level by 2.97 mm annually over the past 30 years (1989–2018), which all surpassed the global average (2). In addition, the number of heatwave days is expected to increase 3.5 times at the end of the 21st century when greenhouse gas emissions are released as the current trend (3). In Korea, coupling trends of population aging and climate change are making the entire society more vulnerable to climate change. The natural disasters incurred by climate change increase the number of deaths and illnesses and damage the health of vulnerable populations, including older people in Korea (4). The heatwave scenario forecasted that the number of older adults exposed to heatwaves in Korea would increase 3.8 to 5.5 times by 2060 compared to 2020, which is significant compared to the impact on general population, which is about 1.2 to 1.7 times (5).

Older adults are particularly vulnerable to climate-related risks due to physiological frailty, coupled with socioeconomic factors. They are more susceptible to extreme weather due to a decreased ability to control body temperature and an increased risk of chronic diseases (6). The older adults in Korea who are in poverty or living alone struggle more to respond quickly to climate-related risks (7, 8). Notably, 32.8 and 38.2% of the total older adult population in Korea were living alone and in poverty, respectively (9, 10). Also it has been reported that communities with an increasing older adult population tend to limit investment in green infrastructure, which supports the climate resilience of the residents, due to increased health and welfare-related spending (11).

In response to the forthcoming and growing impact of the combination of aging and climate change to older adults and the aging society, the Korean government has taken several policy actions. Since the first plan was published in 2011, the 5-year National Climate Crisis Adaptation Plans have consistently prioritized the protection of older adults as a population vulnerable to the impacts of climate change. In particular, the national plan considered both aging and climate change together and involved the ‘whole of government approach’, engaging multiple sectors and ministries in coordinated action. Also, partial components of national disaster management or healthcare plans addressed the intersection of aging and environment, mostly in response to extreme weather. The Ministry of Health and Welfare recently developed healthy city indices in 2022 that reflected population aging and climate change trends.

With population aging and climate change as pressing policy concerns in Korea, it is important to clarify how these challenges are reflected in existing countermeasures and practices. However, there is currently no comprehensive review of interventions in Korea addressing the nexus of aging and climate change. Hence, this article aims to map the interventions in Korea promoting age-friendly environments aligning with or complementing climate change adaptation and mitigation efforts. At the global level, a review highlighted similar evidence gaps on healthy aging interventions related to climate change in older adults. Building on this, our study contributes to filling this gap by focusing on interventions that have been implemented in Korean cities and communities, providing country-specific insights into how policy intentions are translated into practice (12). In Korean settings, prior studies provide incomplete coverage of our research question. A review on environments for older adults identified that existing evidence largely concentrated on physical and social factors, with climate change not considered (13). One study examined awareness of connections between climate change and health, yet it did not incorporate the aging dimension (14). Another study on the health impacts of climate change noted that research evaluating the effectiveness of policies and interventions addressing climate change-related disasters remains limited (15).

## Methods

### Search strategy

Two researchers (HKa and JO) conducted this scoping review. Adhering to the Preferred Reporting Items for Systematic Reviews and Meta-Analyses extension for Scoping Reviews (PRISMA-ScR) reporting guidelines, the search was conducted on June 3rd, 2024, using three Korean search engines: DBpia, KCI, and RISS (Supplementary material S1). Due to the algorithms of the search engines, the search collected journal articles and grey literature altogether.

The search strategies for each database were built based on the PCC(Population, Concept, Context) framework (16). Search terms implying older adults and any types of intervention were used for population and context criteria, respectively. Due to limited citations available with direct keywords of environmental sustainability and climate resilience, broader search terms were used for concept criteria, including synonyms relevant to age-friendly cities and communities(AFCC). Search terms were limited to Korean language. Full search strategy can be found in Supplementary material S2.

### Study selection

All retrieved records were imported into EndNote for reference management. In the identification stage, duplicate records were automatically removed using EndNote and any remaining duplicates were manually removed by one researcher (HKa). Once duplicates were removed, screening based on title and abstract according to inclusion and exclusion criteria was conducted. Full-text of the records were subsequently assessed for eligibility.

The review included both peer-reviewed journal articles and grey literature. Theses, dissertations, news articles, books, and conference papers were excluded. There were no restrictions in the publication year, and only Korean-language publications were included in the searches. PCC framework was used to construct clear eligibility criteria. For population criteria, we included articles and reports that apply the aging trend or involve older adults. For concept criteria, we included interventions contributing to making cities and communities healthier addressing environmental sustainability or climate resilience. Two researchers (HKa and JO) used information that can be retrieved from the reviewed publications, while using additional sources to complement, in case more detailed information is needed to decide eligibility. However, we excluded articles centered on the creation or commercialization of technologies in the absence of implementing them in cities and communities. More than one intervention record from the same publication were consolidated as the same one only when both the implementation level (national/si/gun/gu) and the content were identical or very similar. If intervention records were addressed in different publications, they were treated as distinct interventions unless they are identically implemented in the same cities and communities. Policies, programs, projects, and cases already in place or practiced in Korea were included for context criteria. Interventions designed only for research purposes that do not have continuity in cities and communities were excluded. Two researchers (HKa and JO) cross-checked to include the studies that satisfied the criteria. Full study selection criteria can be found in the Supplementary material S3.

### Data charting/extraction

The following detailed information was extracted and categorized from the included studies: title, author, publication year, population, concept (matching WHO Age-Friendly Cities domains), context (intervention name, intervention site in practice, level of initiative the intervention came from). Two researchers (HKa and JO) cross-checked data extraction and categorization. Discrepancies were resolved through discussions between them until a consensus was reached. Records selection, screening, and data extraction were completed by 20 December 2024. Data were tabulated in a Microsoft Excel spreadsheet and subsequently summarized according to the Population, Concept, and Context components of the PCC framework.

## Results

**Figure 1:**
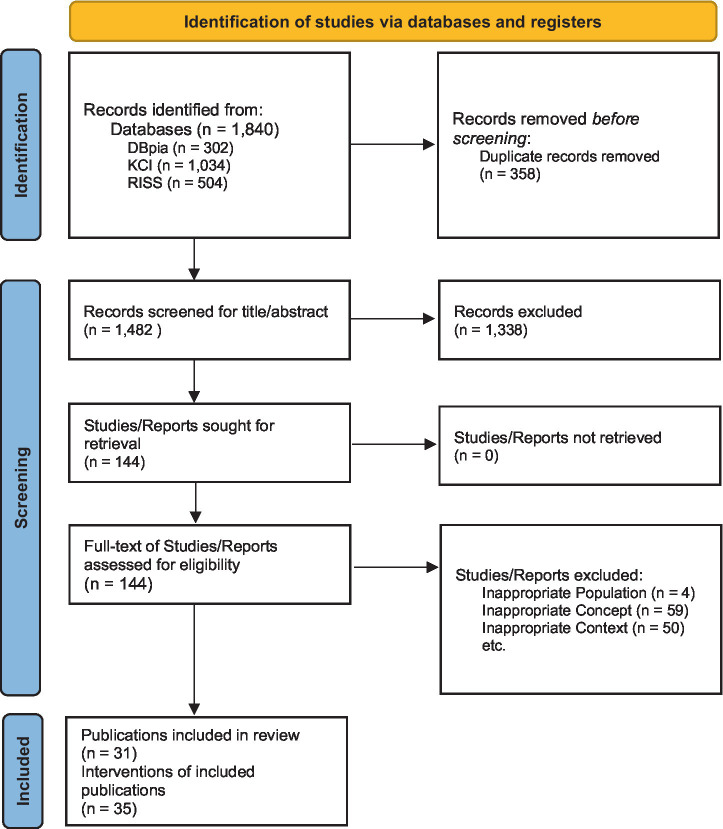
PRISMA flow chart. Adapted with permission from (70).

In total, we have identified 1840 hits from the preliminary search. After deleting 358 duplicates, we screened 1,482 records for title and abstract. After title and abstract screening, 144 publications were retrieved. Thirty-one publications were driven in total after a full-text assessment of 144 records. Then we extracted 35 intervention-level records from the 31 included studies. Because several studies reported multiple eligible interventions, one study contributed three records (17) and six studies contributed two records each (18–23). Following extraction, four duplicated records were identified and removed, yielding the final set of 35 intervention records for analysis (Figure 1).

### Study characteristics

The included records were published between 2006 to 2023. Of 31 included publications, 18 were peer-reviewed journal articles, while 13 were grey literature, including policy briefs, government documents, and reports. Among 31 publications, 23 were empirical studies and more than half (14/23) were categorized as program and policy evaluation. These 14 records were comprised of formative (5/14), process and implementation (5/14), and outcome(4/14) evaluations, following CDC’s Program Evaluation Framework (24). The five formative evaluation studies presented strategies, agenda items, or a plan to enhance the policy/program/practice (25–29). The five process and implementation evaluation studies included experience of beneficiaries and stakeholders involved in the project or program (30–34). The four outcome evaluation studies reported satisfaction or independence of project beneficiaries as outcome measures (35–38). Of these 14 publications, 10 were journal articles and four were grey literatures. Also, the analytic approaches of these 14 program and policy evaluations varied. Five records used qualitative approaches including interviews of older adults, experts and stakeholders such as disaster helpers and government staffs involved in respective intervention implementation (27, 31–34). Another five used quantitative approaches such as surveys of older adults and experts (28, 29, 36–38), and four publications used mixed approaches of both qualitative and quantitative (25, 26, 30, 35). The remaining nine empirical publications were comprised of six descriptive assessment studies (17, 20, 22, 39–41), one measurement and validation study (23), and two modeling studies (5, 42). Seven out of nine were journal articles and two were grey literature. The descriptive assessment studies, presenting current landscape of the practice, program and policy based on data analyses, used mostly (5/6) qualitative data (17, 20, 39–41), whereas one used mixed approach using both quantitative and qualitative data (22). The rest eight out of 31 publications were non-empirical studies which were mostly (7/8) commentaries on policies and programs, describing and reviewing policy, program, and practice to propose policy actions without data analyses (18, 19, 21, 43–46). The majority of non-empirical studies (7/8) were grey literatures (18, 19, 21, 43–45, 47).

Dissecting at the intervention level, sources to retrieve 35 included interventions were distributed similar to publication level: 20 from journal articles and 15 from grey literature; 25 from empirical and 10 were non-empirical studies. Among 25 interventions retrieved from empirical studies, 12 interventions were analyzed in program and policy evaluation: four in formative, 5 in process and implementation, and three in outcome evaluation studies. A full description of the included studies and interventions is identified in Table 1 and the Supplementary material S4.

**Table 1:** Included studies and interventions under the age-friendly cities framework.

Author (publication year)	Intervention	Level of intervention	Cluster 1 (physical environment)			Cluster 2 (social environment)			Cluster 3 (municipal services)	
			D1 (outdoor)	D2 (transport)	D3 (housing)	D4 (social participation)	D5 (employment)	D6 (social inclusion)	D7 (information)	D8 (Health)
Chae (2021) (27)	Visiting home healthcare project	Central								O
Lee (2022) (26)										
Chang (2017) (28)	Healthcare project for climate change vulnerable populations at the health center level	Central							O	O
Cho (2021) (46)	(Local) Heat wave response policy	Local (lower)	O							O
Choi (2012) (45)	Rural Health and Longevity Village Project	Central	O							
Eum and Yun (2015) (32)	(Local) Heat wave response policy	Central	O						O	O
Han (2022) (41)	Smart bus stops	Local (lower)	O	O					O	
Jang (2009) (44)	(National) Heat Wave Response Policy	Central	O						O	O
Shim (2019) (5)										
Jang and Lee (2019) (34)	(Local) Heat Wave Response Policy	Local (upper)	O						O	O
Jang and Kang (2023) (49)	Age-friendly cities project	Local (upper)	O							
Jeon and Lee (2017) (42)	Free shuttle bus	Local (lower)		O						
Kang (2023) (17)	Aging-friendly playgrounds	Local (lower)	O			O				O
	Intergenerational playgrounds	Local (upper)	O			△		△	△	O
	Aging-friendly Playgrounds	Local (upper)	O			O				O
Lim (2023) (22)										
	Older adults-friendly parks	Local (upper)	O			O		O		O
Ki (2008) (19)	Remote protection system for older adults living alone	Local (lower)			O				O	O
	Remote protection system for older adults living alone	Central			O				O	O
Kim (2013) (20)	Transportation safety project for older adults	Local (upper)	O							
	Safe environment project for vulnerable populations	Local (lower)	O							
Kim (2019) (23)	Older adults-friendly parks	Local (lower)	O			O		O	O	O
	Older adults-friendly parks	Local (lower)	O			O		O	O	O
Kim (2022) (36)	Age-Friendly Housing project	Local (lower)			O					O
Lee (2021a) (37)										
Ko (2021) (18)	Pedestrian Safety Project for older adults	Local (upper)	O							
	Age-friendly Town	Local (upper and lower)	O	O	O					
Kwon (2022) (30)	Age-friendly custom home project	Mixed of central, Local (upper), Local (lower), and private			O				△	△
Lee (2013) (43)	Older adults-friendly parks	Local(Lower)	O						O	O
Lee and Park (2015) (40)	Age-friendly Town	Private	O							
Lee (2021b) (35)	Age-friendly custom home project	Local (lower)			O					
Lee (2023) (38)	Age-friendly housing project	Local (upper)			O					
Min (2012) (25)	Older adults-led activities	From community				O				
MoLIT (2018) (47)	Pedestrian priority zone project (Pilot)	Central	O							
Shin (2018) (31)	Project centering the village on community centers	Local (not specified)	O			O		O		
SMG (2006) (21)	Older adults-friendly parks	Local (lower)	O			O				O
	Age-friendly recreation facilities	Local (lower)	O			O		O		O
Sung (2017) (39)	Design for older adults	Local (upper)	O						O	
Yoon (2017) (29)	Barrier free (BF) certification	Central	O		O					

O: Any components of the intervention relevant to environmental sustainability or climate resilience satisfy the respective domain; △: Partly satisfy. D1 (Domain1): Outdoor spaces and buildings; D2 (Domain2): Transportation; D3 (Domain3): Housing; D4 (Domain4): Social participation; D5 (Domain5): Civic participation and employment; D6 (Domain6): Respect and social inclusion; D7 (Domain7): Communication and information; D8 (Domain8): Community support and health services.

### Population

Interventions were either age-friendly or older adults-inclusive. Among 35 interventions, 20 (57.1%) were open to general citizens and were still age-friendly or older adults-inclusive. Twelve interventions targeted older adults, and 3 targeted vulnerable populations, including older adults groups. No intervention was gender specific. Some interventions focused on older adults with specific conditions. 5 interventions included or prioritized those living alone, two on those with low-income, and two on those with disabilities.

### Concept

**Figure 2:**
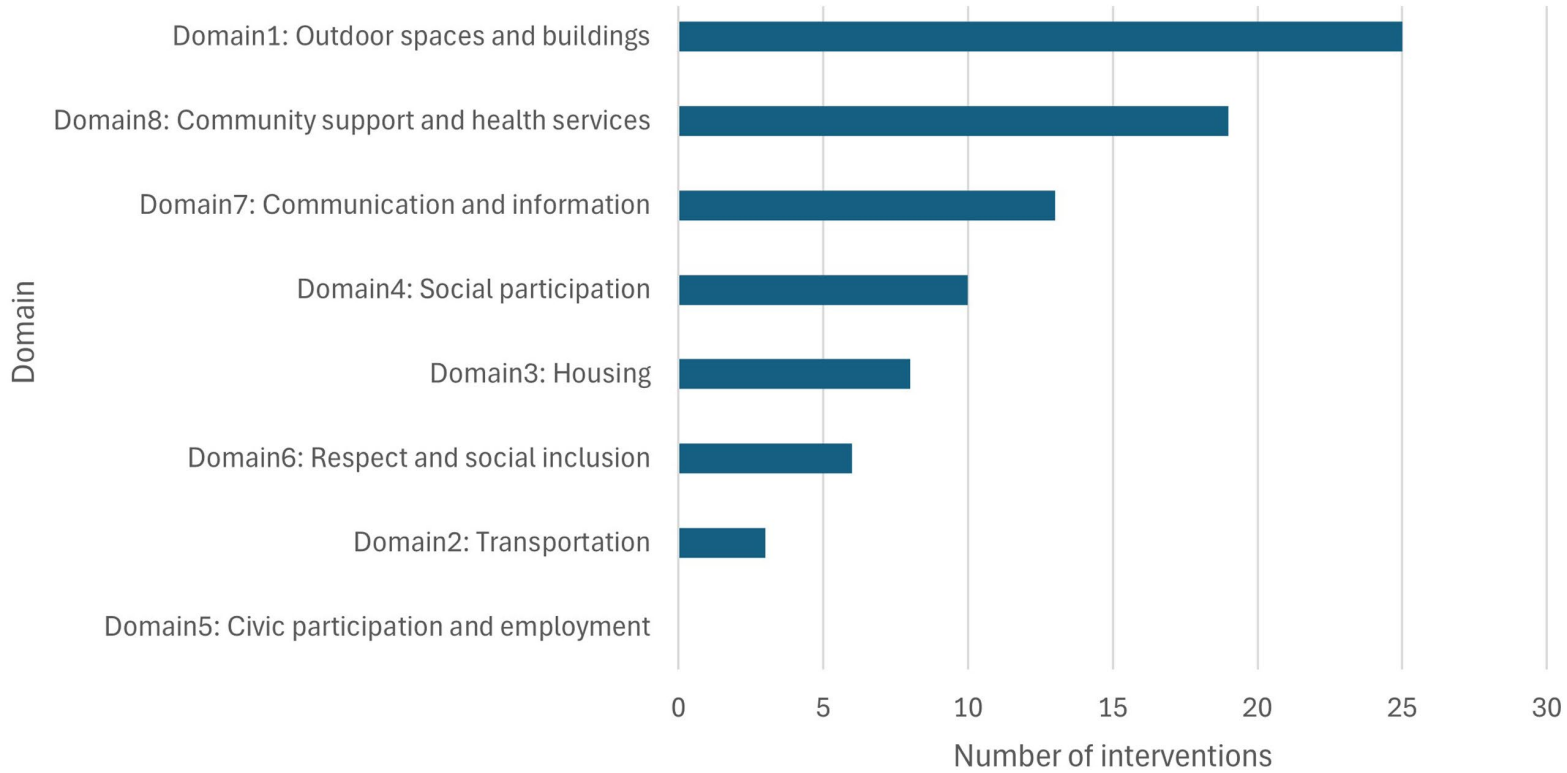
Categorization of included interventions by AFCC domain.

We used the WHO AFCC framework to categorize age-friendly interventions related to environmental sustainability or climate resilience (48). This framework includes eight domains crucial for creating age-friendly environments, grouped into three clusters: Physical environment, Social Environment, and Municipal Service. As a result, interventions included in this study addressed all domains except 5 (Civic participation and employment). Each intervention covers one or more domains at the same time, and domain 1 (Outdoor spaces and buildings) showed the highest frequency (25/35 interventions), followed by 8 (Community support and health services) (19/35 interventions), 7 (Communication and information) (13/35 interventions), 4 (Social participation) (10/35 interventions), and 3 (Housing) (8/35 interventions). The extent to which environmental sustainability or climate resilience was reflected varied by the record, with six records (19.4%) addressing specific climate-related hazards that can be incurred or aggravated by climate change, while the rest showed less clear linkage. Among six interventions, four records were about heatwaves, one about heatwaves and extreme cold, and the last one covered seasonal natural disaster encompassing extreme cold, heatwaves, yellow dust, fine dust, and heavy rain. The distribution of the included interventions in the concept aspect is described in Figure 2.

#### Cluster 1: physical environment

The interventions included in the review primarily focus on the physical environment. The interventions are environmentally friendly or designed to enhance older adults’ access to green elements. Although not directly contributing, they complement or interact with other interventions to contribute to environmental sustainability and climate resilience. Measures against specific climate change-induced risks, such as heat waves and extreme cold, constitute climate-resilient interventions; however, their implementation is often limited to specific seasons of the year.

Outdoor spaces and buildings (Domain 1) match the projects related to safe walking environments (e.g., older adults-conscious signs and barrier-free design) (18, 20, 29, 39, 41, 47), age-friendly indoor and outdoor facilities (cooling shelters, parks, playgrounds, leisure facilities) (5, 17, 21–23, 32, 43, 46, 49), and AFCC projects (18, 20, 31, 40, 45, 49), which encompass both of them. Ensuring safe and accessible walking environments is essential for facilitating older people’s access to age-friendly infrastructure, especially in the context of climate-related risks. The integration of natural elements into outdoor age-friendly infrastructure can enhance environmental sustainability, provided that such spaces are effectively utilized and managed. Furthermore, cities and communities have implemented eco-friendly initiatives that actively engage older people, promoting both social inclusion and environmental responsibility. One study showed cases of age-friendly environments that were self-funded by residents (40).

Under Transportation (Domain 2), the operation of free shuttle buses for older people and smart bus stops plays a vital role in ensuring mobility and safety under extreme weather originating from climate change (18, 41, 42). For example, smart bus stops equipped with air conditioners, air purifiers, UV air sterilizers, solar panels, and heated chairs improve the public transportation environment for all users through inclusive design, while particularly benefiting older adults who are more vulnerable to climate change (41). This underscores the importance of these initiatives in enhancing climate resilience.

Housing (Domain 3) interventions include age-friendly customized housing renovation projects as part of urban regeneration or welfare projects (18, 19, 29, 30, 35–38). These projects aim to improve the housing environment to meet the special needs of older adults with limited mobility and physical conditions. Housing renovations reduced electricity-related financial burdens during extreme summer and winter weather by minimizing drafts and leaks and improving air circulation and were often enhanced independence among highly vulnerable older adults (30, 35). Increased energy efficiency and improved independence of older people can contribute to environmental sustainability and climate resilience (50). A remote protection system enables quick responses in dangerous situations, including climate crises such as heatwaves, especially for single-household older citizens (19). Notably, these projects have been funded either solely by public (18, 29, 30, 35–38) or private sources (30). Many central government-led projects are primarily managed by the Ministry of Land, Infrastructure and Transport as part of the urban regeneration plan (30), and most of the local government-led initiatives were part of community care projects that consider aging-in-place (18, 30, 36, 37). In addition to government-led projects, there were also private actor-led projects. Hospitals implemented housing projects to facilitate patients’ safe transition and sustained residence in their homes (30). There were no interventions observed with the blended funding sources.

#### Cluster 2: social environment

In Korea, age-friendly social environment interventions that include environmentally sustainable or climate-resilient content are often based on natural outdoor facilities and infrastructure earlier addressed in the physical environment cluster. Social participation (Domain 4) occurs based on age-friendly parks, playgrounds, leisure facilities, and community activities in towns or villages (17, 21–23, 25, 31). The case of the age-friendly leisure facility especially shows environmental sustainability as it was recycled from the closed school building (21). In addition, gathering activities initiated and run by older people include gardening and a recycling interest group (25, 31), which are expected to raise awareness and autonomy of older individuals on environmental sustainability. In this review, interventions that fulfill respect and social inclusion (Domain 6) are also characterized by the social participation aspect because community-based activities or activities based on the space of integrated playgrounds lead to intergenerational exchanges (17, 21–23, 31). In particular, trust and cohesion between generations are built through community-based activities (31), which are expected to contribute to community resilience in response to climate change.

#### Cluster 3: municipal services

In the review, Communication and Information (Domain 7) (5, 17, 19, 23, 28, 30, 32, 34, 39, 41, 43, 44) and Community support and health services (Domain 8) (5, 17, 19, 21–23, 26–28, 30, 32, 34, 36, 43, 46) often appear together in services that utilize outdoor facilities or respond to climate change risks. Older people can improve their physical fitness in parks and playgrounds by exercising and get information through bulletin boards and interpersonal communication (17, 21–23, 43). In situations such as cold and heat waves, door-to-door health care, intensive care for the vulnerable, and remote care services can provide health care and information on how to respond to climate change risks (5, 19, 26, 28, 32, 34, 44, 46). This is done through education and behavioral training, enhancing older adults’ response capacity. Establishing age-friendly information design, such as signage design with a unified font system (39), falls under the Communication and information domain and contributes to delivering clear information to older people, which promotes the use of the community infrastructure and service when needed, especially at climate change risk. Some age-friendly housing projects were part of enhancing care services for older adults (30, 36, 37).

### Context

Of 35 interventions, 29 were led by governments initiatives or projects, 23 and 6 from local and central government, respectively. One intervention was initiated and run by the private sector, while two interventions resulted from collaboration between government and private agencies. One intervention was developed from a small number of older people in the community with the help of an NGO. The site of the intervention taken in place varied. However, more than half of the interventions (21/35) occurred in the Seoul Metropolitan Area. Also, 18 interventions had more than one composition of activities with different characteristics, while 17 interventions were composed of single or similar activities.

## Discussion

### Summary of evidence

In our review, we identified the existing evidence of age-friendly interventions implemented in Korean cities and communities which concerned climate change. To the best of our knowledge, this is the first scoping review of Korea’s interventions in the intersection of aging and climate change.

Few age-friendly interventions in Korean cities and communities adequately incorporate the impacts of climate change. Although we found a wealth of literature tackling either aging or climate change throughout the review, only 35 interventions from 31 publications addressed both simultaneously. Environmental sustainability or climate resilience were not comprehensively addressed in the reviewed interventions, though they were partially reflected in the green or nature-friendly aspects. The reviewed interventions related to or contributing to environmental sustainability or climate resilience were largely skewed toward specific AFCC domains such as domain 1(Outdoor spaces and buildings) and 8(Community support and health services). Except for responses to extreme weather, where there are feedback loops that governments have developed interventions over a long period, the physical environment and environment-friendly activities were dominant. This is consistent with another Korean study suggesting that awareness of the health impacts of climate change remains relatively low and systematic response strategies are insufficient, although the study did not focus specifically on age-friendly environments (14). An earlier review of climate change adaptation research in Korea similarly highlighted the inadequate integration of climate change adaptation policies into health and welfare (51).

Interventions addressing the same climate-related hazard were found to span multiple AFCC domains, either individually or in combination. Nonetheless, explicit specification of the targeted climate-related hazards was largely absent, except for temperature-related risks such as heatwaves and extreme cold. Considering evidence-based policy and practices are based on existing evidence, it is consistent with a review finding of climate hazard impacts on older adults that existing evidence are predominantly limited to ambient temperature-related hazards (52, 53). Some interventions appeared to generate spill-over benefits beyond their primary hazard focus, potentially reducing older adults’ vulnerability to other hazards—such as fine dust and seasonal heavy rain—by enhancing their mobility, energy security, social inclusion, and engagement in outdoor activities. Although these hazards were discussed in discrete contexts, they are underpinned by common vulnerabilities among older adults. Therefore, incorporating multi-hazard strategies within the WHO AFCC framework may offer a more coherent and sustainable pathway to strengthening both environmental sustainability and climate resilience in aging cities and communities. Consistent with this observation in Korean context, a recent review similarly found that most global interventions addressing aging and climate change remain fragmented and largely focused on vulnerability and disaster response (12).

This review revealed that critical areas such as civic participation and employment opportunities for older adults remain insufficiently addressed in the context of climate change. In Korea, ongoing discourse and policy efforts have emphasized employment of older adults and their social participation, driven by both their willingness to remain economically active and their financial needs (54, 55). While the government operates a wide range of employment and activity support programs for older people, including both public and private sector models, environmental sustainability and climate resilience were not addressed initially in their designs. This gap is compounded by weak empowerment among older adults, limiting the emergence of best practices in civic participation and employment in the context of climate change. A study of older populations in small and medium-sized Korean cities found that they placed more responsibility for climate adaptation on governments than on individuals (56). Some grassroots efforts are emerging in 2022, a climate change advocacy group run by older adults was launched in Korea, inspired by the 'Grey and Green' movement in Europe. However, such instances are not yet diverse or widespread (57).

We also found that most interventions were government-driven. A significant portion of interventions in infrastructure have been driven by government initiatives, particularly those that focus on improving accessibility and creating age-friendly public spaces. Only one record of age-friendly housing renovation was privately funded by the residents (38). The smart bus stop interventions were implemented and funded by the government, although the idea introduction and technology application were initially developed through cooperation between the government and the private sector (41). The notably limited mention of non-governmental sector engagement in this review may be linked to the characteristics of Korea's governance structure. Although collaborative efforts between government and non-government entities do exist, many are small in scale or remain underreported or underrecognized. Previous studies have also highlighted that Korea’s centralized policy-making structure hinders effective responses to climate change at the local and community levels. The dominance of central government-led planning and authorities, limited participation from local governments and non-state actors (e.g., private enterprises, civil society organizations, and local communities) and insufficient fiscal and administrative decentralization have collectively impeded the identification and development of locally grounded climate adaptation and mitigation actions (58–60). Recognizing and strengthening the role of non-governmental stakeholders in the WHO AFCC framework could guide many countries in developing more innovative and sustainable approaches to building climate resilience among older adults.

Lastly, the indicators used in most evidence to assess the interventions were very limited. 12 interventions from 14 publications that evaluated the intervention addressed different indicators depending on the stages of evaluation, yet scarcely included aspects of environmental sustainability or climate resilience as a common characteristic. Especially those eight interventions retrieved from outcome and process and implementation evaluation studies presented independence, satisfaction, and experience of the older adults or stakeholders but did not contain survey questionnaires or interview items that considered environmental sustainability or climate resilience. The lack of diversity and limitations of applicability of environmental sustainability or clinical resilience identified through our study are also supported by existing research results. In many cases, in studies on the impact of climate hazards on older adults, indicators are presented as all-cause mortality, cause-specific mortality, and morbidities (61). However, these indicators are limited in defining the problem, which presents individual environmental factors and their health effects and suboptimal to assess holistic effectiveness of policy, program and intervention in the nexus of aging and climate change (51).

### Limitations

The study included journal articles and grey literature such as policy briefs, government documents, and reports. However, including diverse media formats such as news articles can help the review be more inclusive. The diversity of the environmentally sustainable and climate-resilient countermeasures for an aging population, especially those driven by civil society, private sectors, or lower levels of municipalities, can bring additional insights.

### Recommendations for further practice

Missing or lacking components of age-friendly interventions in practice represent a gap that could undermine the broader effectiveness of Korea’s aging policies, especially with the added challenges posed by climate change.

The lack of comprehensiveness may be attributed to the limited consideration or discussion of environmental sustainability and climate resilience, as priority has been given to addressing the core components of the existing WHO AFCC framework. To fill the gaps, it is imperative to review and incorporate climate change adaptation and mitigation into existing age-friendly interventions. In Korea, the number of employed individuals aged 65 and older, and their employment rate have steadily increased through government-led initiatives supporting their employment and social participation (55). It was also accompanied by an expansion of public-private partnerships (62). In contrast to the growth in quantity and quality, however, climate change risks have yet to be adequately considered. To ensure long-term sustainability and resilience, it is recommended that policymakers incorporate climate-related risks and environmental factors into the planning and implementation of older adult civic participation and employment policies. The older adults in the early stages of later life—often referred to in Korea as “young seniors” can serve as one of the key agents in advancing locally grounded and sustainable adaptation and mitigation efforts by promoting community engagement. Leveraging existing participatory platforms may offer a practical pathway for such engagement. This aligns with the importance of local community champions in promoting and sustaining age-friendly approaches (63). For example, the Seoul Metropolitan Government operates an “Older Adults Policy Monitoring Group”, which channels the voices and needs of older citizens into the formulation and implementation of age-friendly policies and programs (64). It is similar to the Rhode Island and Healthy Ageing project in UK that included older adults identifying priorities for specific communities (12).

The predominance of government-led interventions has contributed to a consistent and uniform national approach, playing a pivotal role in scaling age-friendly initiatives and ensuring alignment with broader policy frameworks. While these top-down efforts have been instrumental, government and non-government actors offer distinct yet complementary strengths in advancing such interventions. It is also emphasized in previous evidence that the bottom-up approach was essential to identify the needs of individual older adults and their communities and substantial local capability positions this approach to fit into heterogeneous local structure (63). Expanding the involvement of civil society and the private sector can foster greater innovation, responsiveness, and contextual adaptation. Collaboration and partnership in different stakeholders have been observed to contribute to development, implementation and sustainment of age-friendly programs and initiatives (63). Examples of smart bus stops, smart well-being monitoring service, and AI-based care service in Korea demonstrate how collaborations between private sector with technological capacity and local government with administrative coverage have subsequently been scaled broadly (65–67). As another example, Seoul metropolitan government collaborated with convenience store chains that operates 24/7 to run cool shelters to enhance accessibility (68). This collaborative models underscore the importance of further exploring the roles of non-governmental stakeholders, whose engagement can enhance both the effectiveness and local relevance of age-friendly initiatives.

Although limited in number, existing studies on age-friendly interventions addressing climate change lack robust evaluation indicators, hindering holistic evidence-based policy development in Korea. The Korean government’s report—such as in the Enhanced Third National Climate Crisis Adaptation Plan—summarizes progress but tend to use piecemeal and short-term indicators, leading to deliver limited insight in the intersection of climate and health. Globally, there are high-level indicators suggested in the intersection of climate change on health, composed of adaptation, planning, and assessment section that assess the plans and capacities of national and local level, and adaptation delivery and implementation section which includes indicators on climate information for health, green space, and air conditioning benefits and harms (69). However, these suggested indicators may be instrumental at government level to monitor but may not be as salient at the community level at which policy, program and intervention is expected to run through iterative loop to advance and adapt to city and community context and their changing trends. To develop implementation indicators, interdisciplinary implementation research should be promoted to generate empirical evidence on how aging and climate change intersect in community interventions. it can also help advance a more holistic understanding of outcomes.

### Conclusion

This scoping review shows there is limited evidence of age-friendly policies and interventions implemented in Korea concerning climate change, particularly in environmental sustainability and climate resilience context. Given Korea’s dynamic policy environment, marked by successful local initiatives that often scale to national levels, the combination of strong central government leadership and proactive local government efforts plays a vital role in addressing city and community-specific challenges. Hence, it is recommended to incorporate climate change discussions, particularly into the neglected AFCC domains, through community engagement, to involve diverse stakeholders, including civil society and the private sector, and to support long-term monitoring, evaluation, and related research. Future research should examine practical integration of climate change strategies into Korea’s age-friendly policies, with attention to intersectoral collaboration and its impacts on health equity and community resilience.

## Data Availability

The original contributions presented in the study are included in the article/Supplementary material, further inquiries can be directed to the corresponding author.
