# Correction: Interventions for environmentally sustainable and climate-resilient cities and communities for the aging population in South Korea: a scoping review

**DOI:** 10.3389/fpubh.2026.1878829

**Published:** 2026-05-29

**Authors:** Hyunjin Kang, Jieun Oh, Ho Kim, Siwon Lee, Mikiko Kanda, Pankyu Park, Sally Edwards

**Affiliations:** 1 Institute of Health and Environment, Seoul National University, Seoul, Republic of Korea; 2 Graduate School of Public Health, Seoul National University, Seoul, Republic of Korea; 3 World Health Organization Regional Office for the Western Pacific, Manila, Philippines; 4 WHO Asia-Pacific Centre for Environment and Health in the Western Pacific Region, Seoul, Republic of Korea

**Keywords:** AFCC, age-friendly cities and communities, climate resilience, environmental sustainability, Korea, older adults, healthy aging, healthy city

In the published article, there was a mistake in the affiliations of some authors. The affiliations of all authors in the original article were displayed as:

Hyunjin Kang^1†^, Jieun Oh^2^, Ho Kim^1, 2*^, Siwon Lee ^3^, Mikiko Kanda^3^, Pankyu Park^3^ and Sally Edwards^3^

^1^Institute of Health and Environment, Seoul National University, Seoul, Republic of Korea

^2^Graduate School of Public Health, Seoul National University, Seoul, Republic of Korea

^3^WHO Asia-Pacific Centre for Environment and Health in the Western Pacific Region, Seoul, Republic of Korea

The correct version of the affiliations is:

Hyunjin Kang^1†^, Jieun Oh^2^, Ho Kim^1, 2*^, Siwon Lee^3^, Mikiko Kanda^3^, Pankyu Park^3^ and Sally Edwards^4^

^1^Institute of Health and Environment, Seoul National University, Seoul, Republic of Korea

^2^Graduate School of Public Health, Seoul National University, Seoul, Republic of Korea

^3^World Health Organization Regional Office for the Western Pacific, Manila, Philippines

^4^WHO Asia-Pacific Centre for Environment and Health in the Western Pacific Region, Seoul, Republic of Korea

Authors Siwon Lee, Mikiko Kanda, and Pankyu Park were erroneously assigned to affiliation “WHO Asia-Pacific Centre for Environment and Health in the Western Pacific Region, Seoul, Republic of Korea.” This affiliation has now been removed for authors Siwon Lee, Mikiko Kanda, and Pankyu Park.

Affiliation “World Health Organization Regional Office for the Western Pacific, Manila, Philippines” was omitted for authors Siwon Lee, Mikiko Kanda, and Pankyu Park. This affiliation has now been added for authors Siwon Lee, Mikiko Kanda, and Pankyu Park.

The original version of this article has been updated

