# Supplementary material for: Interventions for environmentally sustainable and climate-resilient cities and communities for the aging population in South Korea: a scoping review
Source: Front Public Health. 2026 Apr 13;14:1731172. doi: 10.3389/fpubh.2026.1731172 (PMC13111168; doi:10.3389/fpubh.2026.1731172)
Supplement: Supplementary file 1 [file Supplementary_file_1.docx]

**S1. Preferred Reporting Items for Systematic reviews and Meta-Analyses extension for Scoping Reviews (PRISMA-ScR) Checklist**

| **SECTION** | **ITEM** | **PRISMA-ScR CHECKLIST ITEM** | **REPORTED ON**  **PAGE #(LOCATION)** |
| --- | --- | --- | --- |
| **TITLE** | | | |
| Title | 1 | Identify the report as a scoping review. | 1 |
| **ABSTRACT** | | | |
| Structured summary | 2 | Provide a structured summary that includes (as applicable): background, objectives, eligibility criteria, sources of evidence, charting methods, results, and conclusions that relate to the review questions and objectives. | 1 |
| **INTRODUCTION** | | | |
| Rationale | 3 | Describe the rationale for the review in the context of what is already known. Explain why the review questions/objectives lend themselves to a scoping review approach. | 2 (Introduction) |
| Objectives | 4 | Provide an explicit statement of the questions and objectives being addressed with reference to their key elements (e.g., population or participants, concepts, and context) or other relevant key elements used to conceptualize the review questions and/or objectives. | 2 (Introduction) |
| **METHODS** | | | |
| Protocol and registration | 5 | Indicate whether a review protocol exists; state if and where it can be accessed (e.g., a Web address); and if available, provide registration information, including the registration number. | *No protocol*  *exists* |
| Eligibility criteria | 6 | Specify characteristics of the sources of evidence used as eligibility criteria (e.g., years considered, language, and publication status), and provide a rationale. | 2 (Method -Study selection), Supplementary material S3 |
| Information sources* | 7 | Describe all information sources in the search (e.g., databases with dates of coverage and contact with authors to identify additional sources), as well as the date the most recent search was executed. | 2 (Method -Study strategy) |
| Search | 8 | Present the full electronic search strategy for at least 1 database, including any limits used, such that it could be repeated. | Supplementary material S2 |
| Selection of sources of evidence† | 9 | State the process for selecting sources of evidence (i.e., screening and eligibility) included in the scoping review. | 2 (Method -Study selection, Search strategy) |
| Data charting process‡ | 10 | Describe the methods of charting data from the included sources of evidence (e.g., calibrated forms or forms that have been tested by the team before their use, and whether data charting was done independently or in duplicate) and any processes for obtaining and confirming data from investigators. | 3 (Method -Data charting/extraction) |
| Data items | 11 | List and define all variables for which data were sought and any assumptions and simplifications made. | 3 (Method -Data charting/extraction) |
| Critical appraisal of individual sources of evidence§ | 12 | If done, provide a rationale for conducting a critical appraisal of included sources of evidence; describe the methods used and how this information was used in any data synthesis (if appropriate). | *No critical appraisal*  *implemented* |
| Synthesis of results | 13 | Describe the methods of handling and summarizing the data that were charted. | 3 (Method - Data charting/extraction) |
| **RESULTS** | | | |
| Selection of sources of evidence | 14 | Give numbers of sources of evidence screened, assessed for eligibility, and included in the review, with reasons for exclusions at each stage, ideally using a flow diagram. | 3 (Results), Figure1 |
| Characteristics of sources of evidence | 15 | For each source of evidence, present characteristics for which data were charted and provide the citations. | 4 (Results- Study characteristics) |
| Critical appraisal within sources of evidence | 16 | If done, present data on critical appraisal of included sources of evidence (see item 12). | *No critical appraisal*  *implemented* |
| Results of individual sources of evidence | 17 | For each included source of evidence, present the relevant data that were charted that relate to the review questions and objectives. | 4-8 (Results- Population, Concept, Context, Table1) |
| Synthesis of results | 18 | Summarize and/or present the charting results as they relate to the review questions and objectives. | 4-8 (Results- Population, Concept, Context, Table1) |
| **DISCUSSION** | | | |
| Summary of evidence | 19 | Summarize the main results (including an overview of concepts, themes, and types of evidence available), link to the review questions and objectives, and consider the relevance to key groups. | 8-9 (Discussion- Summary of evidence) |
| Limitations | 20 | Discuss the limitations of the scoping review process. | 9-10 (Discussion- Limitations) |
| Conclusions | 21 | Provide a general interpretation of the results with respect to the review questions and objectives, as well as potential implications and/or next steps. | 10 (Discussion- Conclusion) |
| **FUNDING** | | | |
| Funding | 22 | Describe sources of funding for the included sources of evidence, as well as sources of funding for the scoping review. Describe the role of the funders of the scoping review. | 11 (Funding) |

JBI = Joanna Briggs Institute; PRISMA-ScR = Preferred Reporting Items for Systematic reviews and Meta-Analyses extension for Scoping Reviews.

* Where *sources of evidence* (see second footnote) are compiled from, such as bibliographic databases, social media platforms, and Web sites.

† A more inclusive/heterogeneous term used to account for the different types of evidence or data sources (e.g., quantitative and/or qualitative research, expert opinion, and policy documents) that may be eligible in a scoping review as opposed to only studies. This is not to be confused with *information sources* (see first footnote).

‡ The frameworks by Arksey and O’Malley (6) and Levac and colleagues (7) and the JBI guidance (4, 5) refer to the process of data extraction in a scoping review as data charting*.*

§ The process of systematically examining research evidence to assess its validity, results, and relevance before using it to inform a decision. This term is used for items 12 and 19 instead of "risk of bias" (which is more applicable to systematic reviews of interventions) to include and acknowledge the various sources of evidence that may be used in a scoping review (e.g., quantitative and/or qualitative research, expert opinion, and policy document).

*From:* Tricco AC, Lillie E, Zarin W, O'Brien KK, Colquhoun H, Levac D, et al. PRISMA Extension for Scoping Reviews (PRISMAScR): Checklist and Explanation. Ann Intern Med. 2018;169:467–473. [doi: 10.7326/M18-0850](http://annals.org/aim/fullarticle/2700389/prisma-extension-scoping-reviews-prisma-scr-checklist-explanation).

**S2. Search strategy**

| **#1** | |
| --- | --- |
| **Name** | **RISS**(Research Information Sharing Service) |
| **Publisher** | KERIS (Korea Education & Research Information Service) |
| **Date** | 2024-06-03 |
| **Search String (Korean)** | 전체 : "고령"\|"노령"\|"노인"\|"어르신"\|"실버"\|"시니어" <AND> 전체 : "고령친화"\|"친고령"\|"지속가능"\|"회복탄력"\|"기후탄력"\|"기후회복"\|"건강도시"\|"도시건강"\|"건강마을" <AND> 전체 : "정책"\|"프로그램"\|"사업"\|"중재"\|"사례" |
| **Search String (English)** | Total : “aged”\|"aged"\|"older adults"\|"senior citizens"\|"silver"\|"senior" <AND> Total : "age-friendly"\|"age-friendly"\|"sustainable"\|"resilient"\|"climate-resilient"\|"climate-resilient"\|"healthy city"\|"urban health"\|"healthy village" <AND> Total : "policy"\|"program"\|"project"\|"intervention"\|"case" |
| **Total number** | **504** |

| **#2** | |
| --- | --- |
| **Name** | **DBpia** |
| **Publisher** | Nurimedia Co., Ltd. |
| **Date** | 2024-06-03 |
| **Search String (Korean)** | 전체="고령"\|"노령"\|"노인"\|"어르신"\|"실버"\|"시니어" AND 전체="고령친화"\|"친고령"\|"지속가능"\|"회복탄력"\|"기후탄력"\|"기후변화"\|"건강도시"\|"도시건강"\|"건강마을" AND 전체="정책"\|"프로그램"\|"사업"\|"중재"\|"사례" |
| **Search String (English)** | Total="aged"\|"aged"\|"older adults"\|"senior citizens"\|"silver"\|"senior" AND Total="age-friendly"\|"age-friendly"\|"sustainable"\|"resilient"\|"climate-resilient"\|"climate-resilient"\|"healthy city"\|"urban health"\|"healthy village" AND Total="policy"\|"program"\|"project"\|"intervention"\|"case" |
| **Total number** | **302** |

| **#3** | |
| --- | --- |
| **Name** | **KCI (Korea Citation Index)** |
| **Publisher** | NRF (National Research Foundation of Korea) |
| **Date** | 2024-06-03 |
| **Search String (Korean)** | KEYALL:("고령"\|"노령"\|"노인"\|"어르신"\|"실버"\|"시니어") AND KEYALL:("고령친화"\|"친고령"\|"지속가능"\|"회복탄력"\|"기후탄력"\|"기후변화"\|"건강도시"\|"도시건강"\|"건강마을") AND KEYALL:("정책"\|"프로그램"\|"사업"\|"중재"\|"사례") |
| **Search String (English)** | KEYALL:("aged"\|"aged"\|"older adults"\|"senior citizens"\|"silver"\|"senior") AND KEYALL:("age-friendly"\|"age-friendly"\|"sustainable"\|"resilient"\|"climate-resilient"\|"climate-resilient"\|"healthy city"\|"urban health"\|"healthy village") AND KEYALL:("policy"\|"program"\|"project"\|"intervention"\|"case") |
| **Total number** | **1034** |

**Note:** Search String (Korean) indicates a combination of search terms used in the study. Search String (English) is a translated version of Search String (Korean) in English. Search terms shown in the Search String (English) include duplicated terms due to one English word matching multiple Korean words.

**S3. Eligibility Criteria**

|  | **Inclusion** | **Exclusion** |
| --- | --- | --- |
| **Population** | - Applying ageing trend or involving senior citizens |  |
| **Concept** | - Interventions contributing to make cities and communities healthier addressing climate-resilience or environmental sustainability. | - interventions centered on the creation or commercialization of technologies in the absence of applying them in cities and communities. |
| **Context** | - Policies, program, projects, and cases already in place or practiced in Korea | - Interventions in other countries  - Program/intervention designed for the research that doesn’t have continuity |
| **Others** | - Language in Korean  - Peer-reviewed journal articles, and policy reports published by Korean government, universities, research institutes or civil societies | - Theses/Dissertations  - News articles  - Books  - Conference paper/proceedings |

**S4. Included studies and interventions**

| **Author**  **(Publication year)** | **Publication type** | **Study type** | **Study subtype** | **Analytic approach*** | **Outcome of the study*** | **Intervention** | **Intervention site in practice** | **Level of Initiative** |
| --- | --- | --- | --- | --- | --- | --- | --- | --- |
| Chae (2021) | Grey Literature | Empirical | Program/Policy evaluation (Formative) | Qualitative | agenda items to enhance the policy/program/practice | Visiting Home Healthcare project | Nationwide | Central |
| Lee (2022) | Grey Literature | Empirical | Program/Policy evaluation (Formative) | Mixed | strategies to enhance the policy/program/practice |  |  |  |
| Chang (2017) | Grey Literature | Empirical | Program/Policy evaluation (Formative) | Quantitative | strategies to enhance the policy/program/practice | Healthcare project for climate change vulnerable populations at the health center level | Cheorwon-gun, Gangwon-do, and Uisung-gun, Gyeongsangbuk-do | Central |
| Cho (2021) | Journal Article | Non-empirical | Policy/Program commentary |  | experience of project beneficiaries and stakeholders | (Local) Heat Wave Response Policy | Goyang-si, Gyeonggi-do | Local(Lower) |
| Choi (2012) | Grey Literature | Non-empirical | Policy/Program commentary |  | landscape of policy/program/practice | Rural Health and Longevity Village Project | Not specified | Central |
| Eum and Yun (2015) | Journal Article | Empirical | Program/Policy evaluation (Process/implementation) | Qualitative |  | (Local) Heat Wave Response Policy | Gwanak-gu, Seoul | Central |
| Han (2022) | Journal Article | Empirical | Descriptive assessment | Qualitative | experience of project beneficiaries and stakeholders | Smart bus stops | Seongdong-gu, Seoul, and Bundang-gu, Seongnam-si, Gyeonggi-do | Local(Lower) |
| Jang (2009) | Grey Literature | Non-empirical | Policy/Program commentary |  | experience of project stakeholders | (National) Heat Wave Response Policy | Nationwide | Central |
| Shim (2019) |  |  |  |  |  |  |  |  |
| Jang and Lee (2019) | Journal Article | Empirical | Program/Policy evaluation (Process/implementation) | Qualitative | a forecast informed policy/program/practice | (Local) Heat Wave Response Policy | Daegu Metropolitan City | Local(Upper) |
| Jang and Kang (2023) | Journal Article | Empirical | Program/Policy evaluation (Process/implementation) | Qualitative | landscape of policy/program/practice | Age-Friendly Cities Project | Sejong Special Self-Governing City | Local(Upper) |
| Jeon and Lee (2017) | Journal Article | Empirical | Modeling/Simulation/Optimization | Modeling | experience of project beneficiaries and stakeholders | Free shuttle bus | Gwanak-gu, Seoul | Local(Lower) |
| Kang (2023) | Journal Article | Empirical | Descriptive assessment | Qualitative | landscape of policy/program/practice | Aging-Friendly Playgrounds | Gongju, Chungcheongnam-do | Local(Lower) |
|  |  |  |  |  |  | Intergenerational playgrounds | Kangdong-gu, Seoul | Local(Upper) |
|  |  |  |  |  |  | Aging-Friendly Playgrounds | Gwangjin-gu, Seoul | Local(Upper) |
| Lim (2023) | Journal Article | Empirical | Descriptive assessment | Mixed | landscape of policy/program/practice |  |  |  |
|  |  |  |  |  |  | Older adults-friendly parks | Jung-gu, and Jongno-gu, Seoul | Local(Upper) |
| Ki (2008) | Grey Literature | Non-empirical | Policy/Program commentary |  |  | Remote Protection System for older adults living alone | Seocho-gu, Seoul | Local(Lower) |
|  |  |  |  |  |  | Remote Protection System for older adults living alone | Buyeo-gun, Chungcheongnam-do, Seongnam-si, Gyeonggi-do, and Sunchang-gun, Jeollabuk-do | Central |
| Kim (2013) | Grey Literature | Empirical | Descriptive assessment | Qualitative | landscape of policy/program/practice | Transportation Safety project for older adults | Daejeon Metropolitan City | Local(Upper) |
|  |  |  |  |  |  | Safe environment project for vulnerable populations | Goyang-si, Gyeonggi-do | Local(Lower) |
| Kim (2019) | Journal Article | Empirical | Measurement/Validation | Mixed | landscape of policy/program/practice | Older adults-friendly parks | Nouwon-gu, Seoul | Local(Lower) |
|  |  |  |  |  |  | Older adults-friendly parks | Namdong-gu, Incheon Metropolitan City | Local(Lower) |
| Kim (2022) | Journal Article | Empirical | Program/Policy evaluation (Outcome) | Quantitative | satisfaction of project beneficiaries | Age-Friendly Housing project | Cheongyang-gun, Chungcheongnam-do | Local(Lower) |
| Lee (2021a) | Journal Article | Empirical | Program/Policy evaluation (Outcome) | Quantitative | satisfaction of project beneficiaries |  |  |  |
| Ko (2021) | Grey Literature | Non-empirical | Policy/Program commentary |  |  | Pedestrian Safety Project for older adults | Dongdaemun-gu, Seongbuk-gu, etc., Seoul | Local(Upper) |
|  |  |  |  |  |  | Age-friendly Town | Seongnam-si, Bucheon-si, and Suwon-si, of Gyeonggi-do | Local(Upper and Lower) |
| Kwon (2022) | Journal Article | Empirical | Program/Policy evaluation (Process/implementation) | Mixed | experience of project stakeholders | Age-friendly custom home project | Mixed | Mixed of Central, Local (Upper), Local(Lower), and Private |
| Lee (2013) | Grey Literature | Non-empirical | Policy/Program commentary |  |  | Older adults-friendly parks | Seongbuk-gu, Seoul | Local(Lower) |
| Lee and Park (2015) | Journal Article | Empirical | Descriptive assessment | Qualitative | landscape of policy/program/practice | Age-friendly Town | Gokseong, Jeollanam-do | Private |
| Lee (2021b) | Journal Article | Empirical | Program/Policy evaluation (Outcome) | Mixed | satisfaction of project beneficiaries | Age-friendly custom home project | Seongbuk-gu, Seoul | Local(Lower) |
| Lee (2023) | Journal Article | Empirical | Program/Policy evaluation (Outcome) | Quantitative | independence of project beneficiaries | Age-Friendly Housing project | Jung-gu, Dong-gu, and Gyeyang-gu of Incheon Metropolitan City | Local (Upper) |
| Min (2012) | Grey Literature | Empirical | Program/Policy evaluation (Formative) | Mixed | a plan to enhance the policy/program/practice | Older adults-led activities | Gwanak-gu, Seoul | From Community |
| MoLIT (2018) | Grey Literature | Non-empirical | Manual/Guideline |  |  | Pedestrian Priority Zone project (Pilot) | Seoul Metropolitan City | Central |
| Shin (2018) | Journal Article | Empirical | Modeling/Simulation/Optimization | Quantitative | experience of project stakeholders | Project centering the village on community centers | Busan Metropolitan City (Not specified) | Local(Not specified) |
| SMG (2006) | Grey Literature | Non-empirical | Program/Policy evaluation (Process/implementation) | Qualitative |  | Older adults-friendly parks | Yangcheon-gu, Seoul | Local(Lower) |
|  |  |  |  |  |  | Age-friendly recreation facilities | Dongjang-gu, Seoul | Local(Lower) |
| Sung (2017) | Journal Article | Empirical | Descriptive assessment | Qualitative | landscape of policy/program/practice | Design for older adults | Seoul Metropolitan City and Gyeonggi-do | Local(Upper) |
| Yoon (2017) | Journal Article | Empirical | Program/Policy evaluation (Formative) | Quantitative | strategies to enhance the policy/program/practice | Barrier Free (BF) certification | Busan Metropolitan City | Central |

*Note: Analytic approach is recorded only for empirical studies; Outcome of the study is recorded only for the evaluation studies. In case multiple outcomes exist, the authors presented one that is relevant to our research question
